# Supplementary material for: Patterns of Intron Gain and Loss in Fungi
Source: PLoS Biol. 2004 Nov 30;2(12):e422. doi: 10.1371/journal.pbio.0020422 (PMC532390; doi:10.1371/journal.pbio.0020422)
Supplement: Table S1 — Also available at http://genes.mit.edu/NielsenEtAl/. (4.3 MB ZIP). [file pbio.0020422.st001.zip › NielsenEtAl/html/1220.html]

AN6232.1.NCU08515.1.MG03244.1.FG00637.1


```
 CLUSTAL W (1.82) Multiple Sequence Alignments - Introns Inserted


Sequence 1: NCU08515.1	513 aa
Sequence 2: MG03244.1	514 aa
Sequence 3: FG00637.1	511 aa
Sequence 4: AN6232.1	507 aa
Alignment Length: 514 aa
Number Identitical Residues: 399 aa
Alignment Score (without introns) 16104


MG03244.1 	MADPRESSSYSVLPRIRYNTIGGVNGPLVILDN0VKNPSYNEIVSLTLPDGTTRSGQVLE
NCU08515.1	MADPRVPSSYNVTPRIRYNTVGGVNGPLVILDN0VKFPRYNEIVTLTLPDGTQRSGQVLE
FG00637.1 	MADPRESSSYSVVPRIRYNTVGGVNGPLVILDN0VKFPRYNEIVTLTLPDGTERSGQVLE
AN6232.1  	MADTLDPRMTSVQPRIRYNTIGGINGPLVFLDN0VKFPTYNEIVSLTLPDGTERSGQVLE
          	***.  .   .* *******:**:*****:*** ** * *****:******* *******

MG03244.1 	ARG1SRAIVQ0VFEGTSGIDVKK0TKVEFTGENLKLGVSEDMLGRIFDGSGRPIDKGPKV
NCU08515.1	ARG1NRAVVQ0VFEGTSGIDVKK0TKVEFTGESLKLGVSEDMLGRIFDGSGRAIDKGPKV
FG00637.1 	ARG1DRAVVQ0VFEGTSGIDVKK0TKVKFTGESLKIGVSEDMLGRIFDGSGRAIDKGPKV
AN6232.1  	ARG1NRAIVQ0VFEGTSGIDVKK0TKVEFTNHSLKLGVSEDMLGRVFDGSGRAIDKGPKV
          	*** .**:** ************ ***:**...**:*********:******.*******

MG03244.1 	LAEDFLDINGQPINPYSR0VYPEEMISTGISAIDTMNSIARGQKIPIFSASGLPHNEIAA
NCU08515.1	LAEEYLDINGSPINPYAR0EYPQEMISTGISAIDTMNSIARGQKIPIFSAAGLPHNEIAA
FG00637.1 	LAEDYLDINGSPINPYSR0EYPEEMISTGISAIDTMNSIARGQKIPIFSASGLPHNEIAA
AN6232.1  	LAEDYLDINGQPINPYSR0VYPEEMISTGISAIDTMNSIARGQKIPIFSASGLPHNEIAA
          	***::*****.*****:*  **:***************************:*********

MG03244.1 	QICRQASLVNKEGVTNKGVHDGHEENFNIVFAAMGVNLETARFFTRDFEENGSLERTTLF
NCU08515.1	QICRQAGLVQRQGITNKGVHDGHEENFSIVFGAMGVNLETARFFTRDFEENGSLGRTTLF
FG00637.1 	QICRQAGLVQKQGITNKGVHDGHEENFSIVFGAMGVNLETARFFTRDFEENGSLERTTLF
AN6232.1  	QICRQAGLVKR---PTKDVHDGHEDNFSIVFAAMGVNMETSRFFTRDFEENGSMERVTLF
          	******.**::   ..*.******:**.***.*****:**:************: *.***

MG03244.1 	LNLANDPT2IERIITPRLALTTAEYYAYQLEKHVLVILTDLSSYCDALREVSAAREEVPG
NCU08515.1	LNLANDPT~IERIITPRLALTTAEYYAYQLEKHVLVILTDLSSYCDALREVSAAREEVPG
FG00637.1 	LNLANDPT2IERIITPRLALTTAEYYAYQLEKHVLVILTDLSAYCDALREVSAAREEVPG
AN6232.1  	LNLANDPT2IERIITPRLALTTAEYYAYQLEKHVLVIMTDLSAYCDALREVSAAREEVPG
          	******** ****************************:****:*****************

MG03244.1 	RRGFPGYMYTDLSTIYERAGRVEGRNGSITQIPILTMPNDD~ITHPIPDLTGYITEGQIF
NCU08515.1	RRGFPGYMYTDLSTIYERAGRVAGRNGSITQIPILTMPNDD~ITHPIPDLTGYITEGQIF
FG00637.1 	RRGYPGYMYTDLSTIYERAGRVSGRNGSITQIPILTMPNDD~ITHPIPDLTGYITEGQVF
AN6232.1  	RRGYPGYMYTDLSTIYERAGRVQGRNGSITQIPILTMPNDD1ITHPIPDLTGYITEGQIF
          	***:****************** ****************** ****************:*

MG03244.1 	VDRGLDNRGIYPPINVLPSLSRLMKSAIGEGMTRKDHGDVSNQLYAKYAIGRDAA~AMKA
NCU08515.1	VDRGLHNRGIYPPINVLPSLSRLMKSAIGEGMTRKDHGDVSNQLYAKYAIGRDAA~AMKA
FG00637.1 	IDRALDNRGIYPPINVLPSLSRLMKSAIGEGMTRKDHGDVSNQLYAKYAIGRDAA~AMKA
AN6232.1  	IDRQLYNKGIYPPINVLPSLSRLMKSAIGKGRTRDDHSDVSNQLYAKYAIGRDAA1AMKA
          	:** * *:*********************:* **.**.***************** ****

MG03244.1 	VVGEEALSAEDKLSLEFLEKFERTFINQGPYEARTIYESLDLAWSLLRIYRKDMLNRIPA
NCU08515.1	VVGEEALSNEDKLSLEFLDKFERSFIAQGPYESRTIFESLDLAWSLLRIYRKDMLNRIPK
FG00637.1 	VVGEEALSAEDKLSLEFLEKFERQFISQGQYESRSIYESLDLAWSLLRIYPKELLNRIPA
AN6232.1  	VVGEEALSSEDKLSLEFLEKFERTFINQSAYESRSIFESLDIAWNLLRIYPPHLLNRIPK
          	******** *********:**** ** *. **:*:*:****:**.*****  .:***** 

MG03244.1 	KIINEYYQRTSERKGKAKAQDKPSAPGTSAAGNPAEENLIDA
NCU08515.1	KIIDEFYSRSAADR-KGKGKDKPTTKDTRDTAAPEEENLIDA
FG00637.1 	KVLNEFYQRAAKES---KSKGKARADTEARGQQQTEENLIDA
AN6232.1  	RVLDEFYARSGRKI----PNKDTRDNSVPEQGQSQTADLIET
          	::::*:* *:.        : ..              :**::
```
